# Free-standing graphene oxide membrane works in tandem with confined interfacial polymerization of polyamides towards excellent desalination and chlorine tolerance performance†

**DOI:** 10.1039/d1na00513h

**Published:** 2021-11-19

**Authors:** Subhasish Maiti, Suryasarathi Bose

**Affiliations:** Department of Materials Engineering, Indian Institute of Science Bangalore Karnataka 560012 India sbose@iisc.ac.in

## Abstract

We explored a unique concept in this study to develop a membrane containing a hierarchical porous architecture derived by etching a specific component from a demixed UCST blend as the support layer and a free-standing GO and a polyamide (PA) layer as functional surfaces. To selectively sieve ions and improve chlorine tolerance performance, three different strategies were proposed here. In the first case, the free-standing GO membrane was used as the active layer. In the second case, the free-standing GO was positioned in tandem with the PA layer formed *in situ*. In the third case, GO was added during the formation of the active PA layer *in situ.* The support layer with a gradient in pore sizes (realized by varying the composition in the blends) was fabricated *via* crystallization induced phase separation in a classical UCST system (PVDF/PMMA) and etching out the amorphous component (here PMMA). A gradient in the pore sizes was obtained by rationally stitching the various membranes obtained by varying the blends' composition. Pure water flux and rejection experiments were carried out to evaluate the performance of this composite membrane. This unique strategy resulted in excellent salt rejection (more than 95% for a monovalent ion), improved fouling resistance (more than 85%), excellent dye removal performance (more than 96% for a cationic dye), and outstanding chlorine tolerance performance and antibacterial activity. Thus, this study emphasizes that the free-standing GO membrane's positioning controls the membranes' overall performance.

## Abstract 2

We explored a unique concept in this study to develop a membrane containing a hierarchical porous architecture derived by etching a specific component from a demixed UCST blend as the support layer and a free-standing GO and a polyamide (PA) layer as functional surfaces.

## Graphical abstract

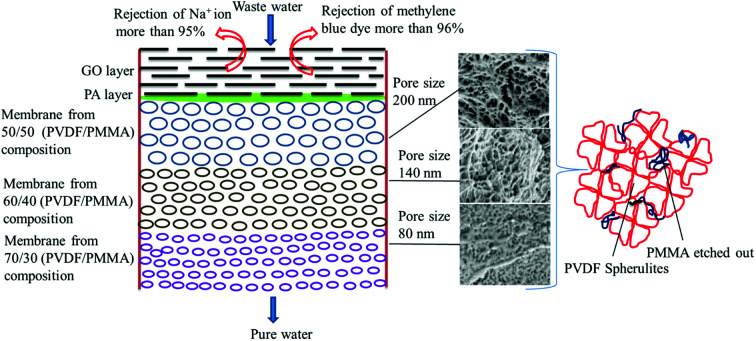

## Introduction

In the 21^st^ century, one of the most significant challenges has been to access safe drinking water. With increasing population and rapid contamination, freshwater aquifers and groundwater levels have been depleting at an alarming rate.^1,2^ So there is an urgent need to reuse unconventional water sources and remediate the contaminated sources.^3^ In this context, desalinating either brackish or seawater seems feasible to sustain the ever-growing population.^3,4^

Among various available techniques like electro-dialysis, evaporation, thermal distillation, and membrane-based distillation, membrane-based distillation is the most economical and practical process.^5,6^ The high cost can be reduced using the Forward Osmosis (FO) technique, which is primarily a membrane separation process driven by natural osmotic pressure, so there is no requirement of a pump.^3^

Although PA-based FO and Reverse Osmosis (RO) techniques are both widely used for desalination, FO is a superior technique as it operates at minimal or no hydraulic pressure without compromising rejection performance when compared with RO based methods. In addition, FO based solutions have also demonstrated the advantage of lower fouling compared to the RO membrane.^7–10^ Various chemical modifications on the surface have been reported in the literature to enhance these membranes' performance. For instance, different carbon-based materials such as carbon nanotubes (CNTs),^11^ carbon dots,^12^ graphene oxide (GO),^13^ graphene oxide quantum dots (GQD),^14^*etc.* have been explored in the recent past. Among these, the GO-based membrane showed some promise as it contains more hydrophilic groups (like carboxyl, hydroxyl and epoxy).^15^ The excellent film-forming ability of GO is due to its good dispersion in aqueous solution and the membrane is formed *via* a layer by layer assembly method.^16^ Moreover, it provides nanochannels within its lamellar structure through which water permeates very quickly. It has been established that incorporating GO into the composite membrane enhances the performance significantly in terms of permeability, antibacterial, antifouling, and chlorine tolerance characteristics.^13,17–21^ As the PA layer is highly susceptible to chlorine attack, which reduces this layer's mechanical strength, the rejection performance is significantly affected. Hence, several surface modifications have been proposed to address this challenge.^22^ To this end, PVDF is used extensively as a membrane material due to its high mechanical strength, chemical resistance, thermal resistance, and excellent film-forming ability. However, like other candidates (PES, cellulose, *etc.*), it suffers from chlorine and fouling attack.

In this work, three strategies were explored for size-selective sieving of ions, improved fouling resistance, and enhanced chlorine tolerance performance. In the first case, the free-standing GO layer was placed on top of the porous support without the PA layer. In the next case, the free-standing GO layer was placed on top of the PA layer, formed *in situ*, and in the third case, GO was mixed with the precursors to form GO + PA *in situ.* A classical UCST system (PVDF/PMMA)^23–25^ was chosen here as the support layer, and by selectively etching one of the blends' components, hierarchical porous structures with a gradient in pore size were obtained. To improve the desalination, antifouling and chlorine tolerance performance, this templated support layer together with a free-standing GO and PA layer in tandem was utilized. This strategy resulted in efficient salt rejection, excellent chlorine tolerance performance, fouling resistance, excellent dye removal performance and antibacterial activity.

## Experimental section

### Materials and reagents

PVDF (Kynar 761) was procured from Arkema and PMMA (876 G) was bought from Gujpol. The details of these materials are listed in our previous work.^26^ Graphene oxide was supplied by Log 9 Materials, Bengaluru, with a lateral dimension of 5 μm and a thickness range of 1–2 nm. 1,3,5-Benzenetricarbonyl trichloride (TMSCl) (98%), acrylic acid (99%), and methylene di-aniline (MDA) were purchased from Sigma Aldrich, and sodium chloride (99.90%), magnesium nitrate (99%), hexane, ethanol, and acetic acid were supplied by a local vendor. All materials and chemicals were used directly without further purification.

### Preparation of blend membranes using crystallization induced phase separation

**Fig. 1:**
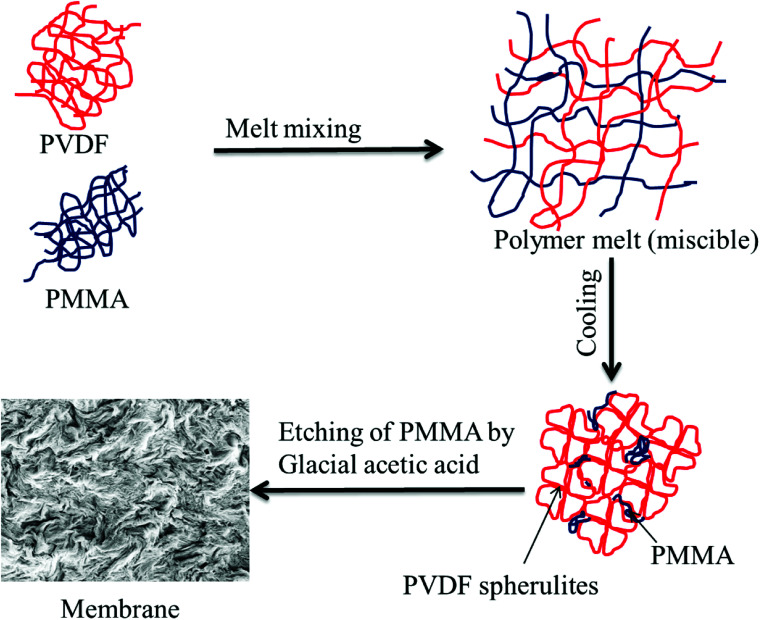
Schematic representation of a membrane fabricated by crystallization induced phase separation.

Blends of PVDF/PMMA with different compositions (50/50, 60/40, and 70/30) were melt processed using a Minilab II HAAKE extruder CTW5 (7 cm^3^) at 220 °C with a 60 rpm screw speed for 20 min as reported in the literature.^23^ Compression-molded discs with 60 mm diameter and 80 μm thickness were made using a laboratory hot press at 220 °C. The membranes were then obtained by selectively etching with glacial acetic acid for 7 days to remove the blends' PMMA phase. A unique hierarchical architecture was obtained by stacking these membranes using PAA solution (0.08 g mL^−1^ THF) (referred to as control membrane hereafter). These were kept for vacuum drying at 40 °C for 24 h. The PAA was prepared in the laboratory by using a free radical polymerization technique. In brief, 10 mL acrylic acid monomer, 5 mg AIBN, and 25 mL THF were added to a 100 mL round bottom flask, and three freeze–pump–thaw cycles were performed. The reaction mixture was then heated at 80 °C under an inert atmosphere for 4 h. The membrane which was fabricated using crystallization induced phase separation is shown in Fig. 1.

### Preparation of free-standing GO

**Fig. 2:**
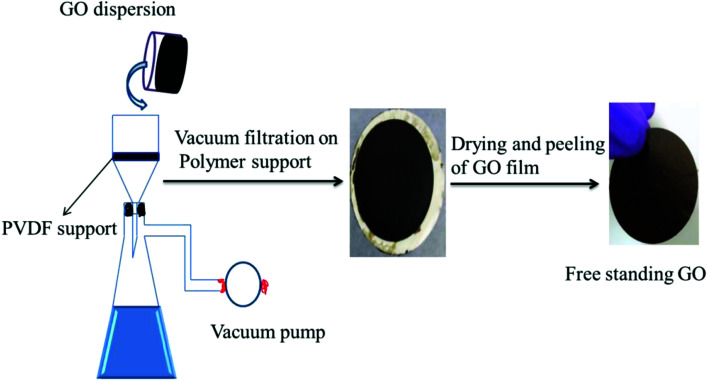
Schematic representation of the fabrication of the free-standing GO membrane by vacuum-assisted filtration using a porous polymer support.

Free-standing GO was prepared by a method reported in the literature.^27^ Typically, 40 mL of deionized water was added to 30 mg of GO and the mixture bath sonicated for 120 min. Then the suspension was deposited onto a commercial hydrophilic PVDF membrane through a vacuum-assisted filtration. The alignment of GO nanosheets was promoted by a prolonged vacuum rate (about 0.1 mL min^−1^). Then the obtained GO membrane was vacuum dried at 60 °C overnight. Finally, it was characterized by various techniques as shown in the ESI.† The schematic representation of the fabrication of free-standing GO is shown in Fig. 2.

### GO–PA thin-film composite membranes

**Fig. 3:**
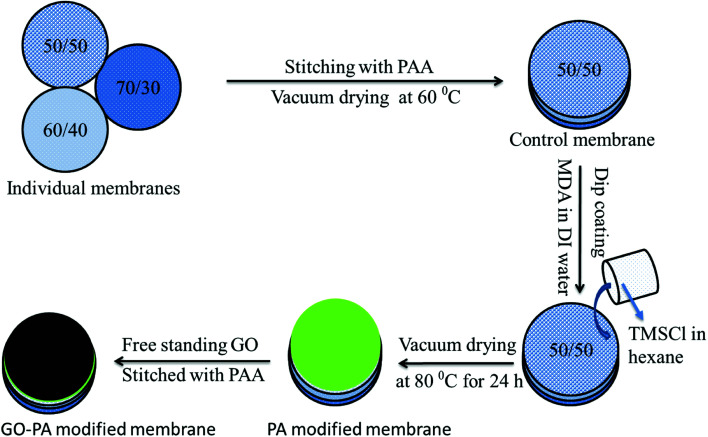
Schematic representation of fabricating the GO–PA modified membrane. 50/50, 60/40 and 70/30 represent the weight% of PVDF and PMMA in the blend, respectively.

Two different types of composite membrane were prepared either by stitching a free-standing GO membrane on the polyamide surface formed *in situ* (referred to as GO–PA modified membrane hereafter) or using GO sheets as a precursor during the formation of the PA layer *in situ* on the active 50/50 layer (referred to as GO + PA modified membrane hereafter). At first, GO and MDA were taken in a 1 : 100 w/w ratio and bath sonicated in a water/ethanol mixture (70/30 v/v) for 30 min. The composite membrane was dipped in that solution and kept for drying at room temperature for 30 min. The amide layer was formed by dip coating the membrane into TMC organic solution, and finally, the membrane was kept at 80 °C for vacuum drying for 24 h. The schematic representation is shown in Fig. 3.

**Fig. 4:**
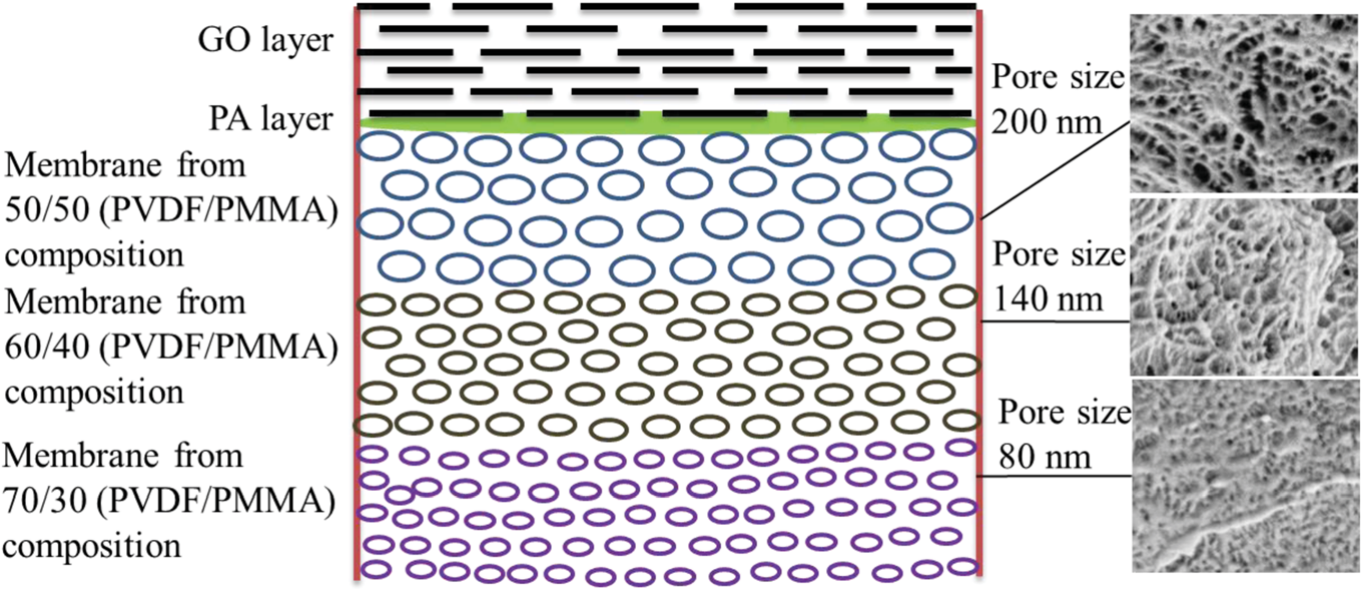
Schematic representation of the gradient in morphology in the GO–PA modified membrane. The corresponding cross-sectional SEM micrographs are shown alongside.

Thus, the GO–PA thin film composite membrane was fabricated by stitching free-standing GO membranes on the polyamide based thin-film composite membranes with varying pore sizes to obtain a gradient in pore morphology. The schematic representation of the gradient in morphology is shown in Fig. 4.

### Membrane characterization

**Fig. 5:**
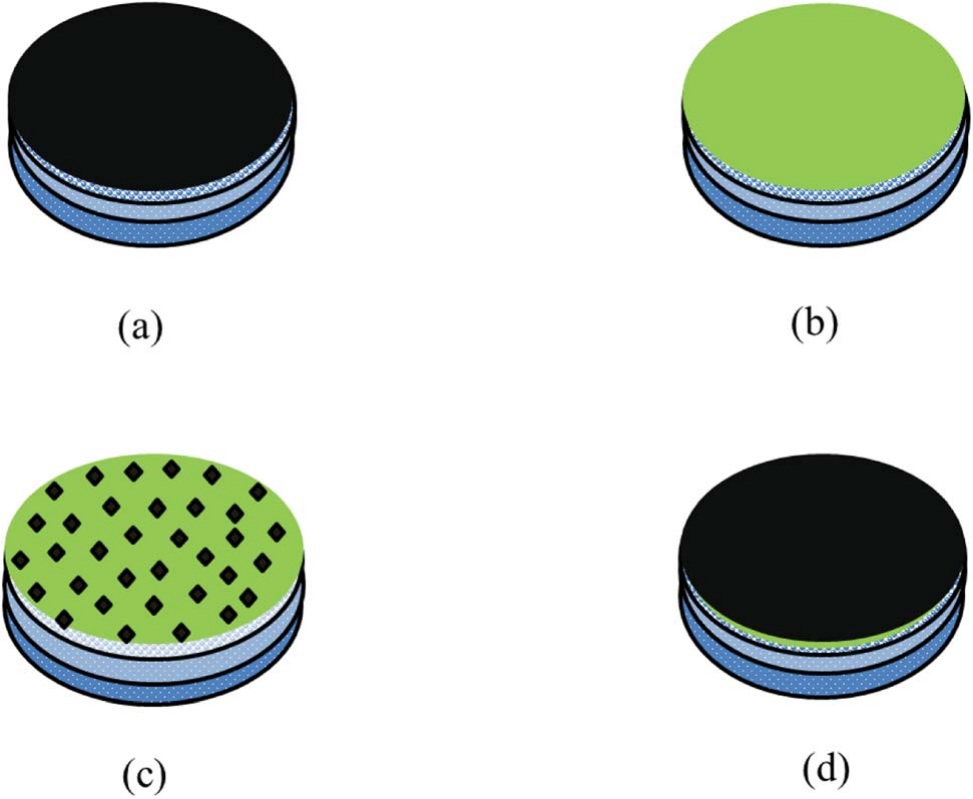
Schematic representation of different composite membranes: (a) GO modified membrane, (b) PA modified membrane, (c) GO + PA modified membrane and (d) GO–PA modified membrane (free-standing GO on the top).

The membranes were characterized by Fourier Transform Infrared spectroscopy (FTIR) performed on a PerkinElmer Frontier in the mid-IR range (4000 cm^−1^ to 650 cm^−1^). The surface and cross-sectional morphologies were characterized using a scanning electron microscope (SEM; Ultra55 FESEM, Carl Zeiss) equipped with an EDX detector. The hydrophilicity of all the modified membranes was determined by contact angle measurement using water as a solvent. The free-standing GO was characterized using XRD. The schematic representation of all the membranes is shown in Fig. 5.

### Permeation assessment and antifouling test

#### (a) Pure water flux

Permeation and separation performances were determined by using a lab-scale in-house cross flow set-up. A piece of each composite membrane of 47 mm dia. was fastened inside the module and compacted at 0.21 MPa for 30 min before reading the data followed by the recording of data by varying the *trans*-membrane pressure from 0.21 MPa to 0.51 MPa. The experiments were carried out in triplicate and repeated with the control membrane without any polyamide layer modification.

#### (b) Dye rejection studies

A cationic dye (MB) was taken as a model dye pollutant for evaluating the dye removal characteristics. The rejection studies were done in the cross-flow mode at 0.41 MPa taking 10 ppm aqueous solution of dye as feed. The concentration of the permeate was estimated using UV Vis spectrophotometry and % rejection was measured using the formula:

1 % Rejection = [1 − ((permeate conc. in ppm)/(feed conc. in ppm))] × 100

#### (c) Salt rejection studies

**Fig. 6:**
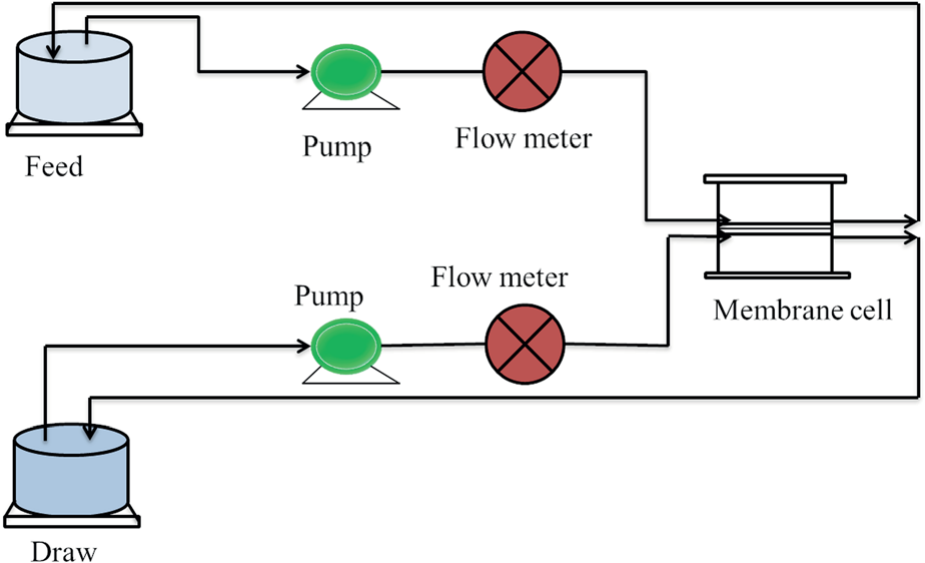
Schematic diagram of the FO lab-scale system.^28^

A monovalent salt, *i.e.* NaCl, was taken as a model draw solution for assessing the rejection performance. The salt rejection performance was evaluated using a FO flux set up designed in the lab as shown in Fig. 6. In this experiment, 1000 ppm NaCl was taken as the draw while double distilled water was used as the feed. The concentrations of the draw and feed were monitored using a TDS meter and the rejection was measured using eqn (2):

2 % Rejection = [1 − ((final draw conc.)/(initial draw conc.))] × 100

#### (d) Dynamic antifouling studies

A dynamic antifouling study was conducted using BSA as a model foulant. As per our previous work,^29^ a concentration of 1 g L^−1^ BSA feed was prepared using double distilled water. DI water flux *J*_w_ as the first step, flux with BSA *J*_B_ as feed, and water flux cycle *J*_P_ after back-flushing the membrane for 30 min in 1× PBS were estimated. The antifouling properties of the membrane were quantified in terms of flux recovery ratio (FRR), irreversible flux decline ratio (IFR), and relative fouled flux ratio (RFR). It is observed that the higher the value of FRR and lower the amount of IFR better is the fouling resistance of the membrane. The FRR, IFR, and RFR were calculated using the following equations.3
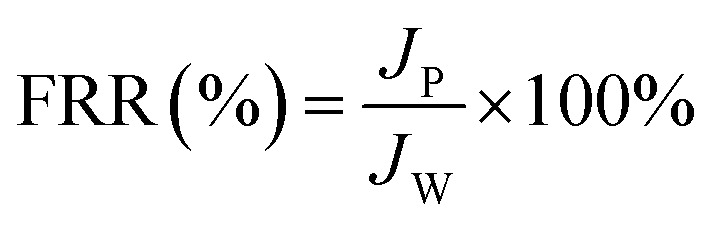

4 IFR (%) = 100 − FRR

5
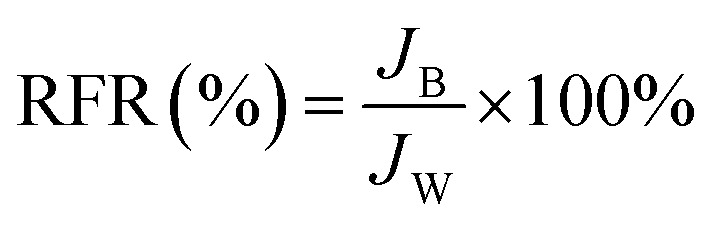
where *J*_w_ = pure water flux, *J*_P_ = water flux after back-flushing with PBS solution, and *J*_B_ = BSA solution flux.

### Chlorine tolerance performance

Membrane chlorine stability was characterized by studying the salt rejection of the membrane before and after exposure to the chlorine solution. The lower the refusal of salt post treating the membrane with a suitable treatment, the better the chlorine tolerability of the membrane. A commercial sodium hypochlorite solution was diluted in double distilled water to reach a concentration of 2000 ppm of pH 10. To investigate the chlorine tolerance property, the control and GO modified membranes were immersed in the solution for 3 h and then these membranes were washed sufficiently with double distilled water. After chlorine exposure, the salt rejection of these membranes was evaluated using a FO experiment like before chlorination using 1000 ppm NaCl aqueous solution as draw and doubled distilled water as feed.

### Bacteriological assessment

The antibacterial activities of the GO–PA modified membrane were examined using model bacterial strains (Gram-positive *S. aureus* ATCC25923 and Gram-negative *E. coli* ATCC25922) through the standard plate count method. The sub-cultures were made in nutrient broth first from master cultures and these were harvested in a shaker incubator at 37 °C. Then a pellet was made by centrifuging the cultures at 3000 rpm and the medium was discarded. The bacterial pellet was thoroughly washed with 1× PBS (phosphate-buffered saline) and re-suspended in PBS to get 10^8^ CFU mL^−1^ for experiments. Then 1000 μL of these suspensions were seeded in a 96 well plate containing tokens of 4.5 mm sample and incubated for two h. Then the supernatant was collected and plated on auger plates using seven-fold serial dilution and the plates were incubated in an incubator for 24 h. The antibacterial efficiency was determined by assessing the colonies. Finally, the samples for SEM imaging were prepared by fixing the membranes with a 4% (v/v) formaldehyde solution.

## Results

### Assessing the porous support with a gradient in pore sizes

**Fig. 7:**
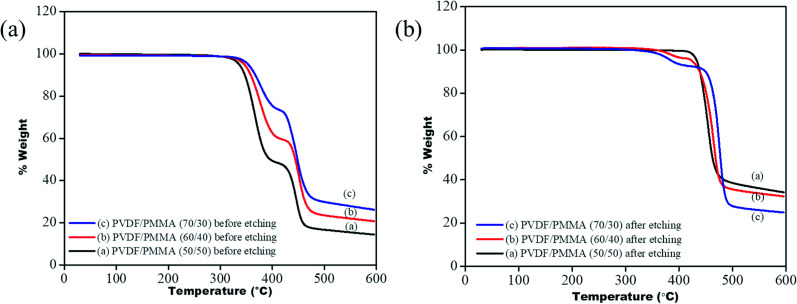
TGA profile of PVDF/PMMA blends (a) before etching (b) after etching.

To ensure the complete removal of PMMA post etching, TGA of all the membrane samples was performed before and after etching using a TA Q500. From the TGA profile (see Fig. 7) it is observed that the blends before etching showed two-step degradation: one corresponding to PVDF, in the range between 420 °C and 480 °C, and another for PMMA, in the range of 320 °C to 400 °C, while a single step degradation is observed for the etched blends, manifesting the complete removal of PMMA. In the case of 60/40 (PVDF/PMMA) blends and 70/30 (PVDF/PMMA) blends, a residual of 6% and 3% PMMA was still present in the system. The portions of un-etched PMMA were probably those segments that were trapped in the inter-lamellar region of PVDF.^22^

### Surface and cross-sectional morphology of individual membranes

**Fig. 8:**
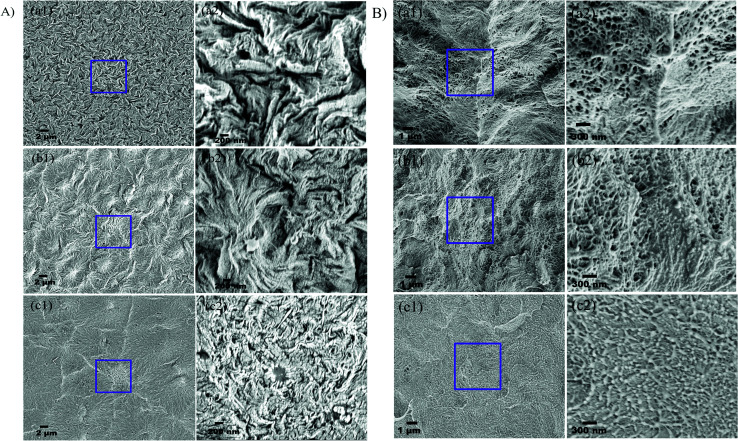
(A) Surface and (B) cross-sectional morphology of PVDF/PMMA blends after etching with glacial acetic acid for 7 days at different compositions: (a) 50/50; (b) 60/40; (c) 70/30.

The surface and cross-sectional morphologies of individual membranes were evaluated using an Ultra55 FESEM from Carl Zeiss. From the SEM micrographs, spherulitic morphology can be observed, as seen in Fig. 8A for the 50/50, 60/40, and 70/30 etched blends, respectively. The spherulites were larger for the combinations containing less PMMA. Fig. 8B shows cross-sectional micrographs of the corresponding surface micrographs. A spongy, porous architecture was perceived, which is responsible for the un-impeded water flow and will be discussed in the subsequent sections. The average pore sizes of the different membranes, *i.e.* 50/50, 60/40, and 70/30, were approximately 200 nm, 140 nm, and 80 nm, respectively, as evaluated using ImageJ software. Thus the pore sizes were controlled by varying the compositions in the blend as confirmed by the SEM micrograph. A Zeiss LSM 880 laser scanning confocal microscopy technique was utilized to confirm the individual membranes' surface and cross-sectional morphology. It also provided a clear idea about pore localization in the respective membranes. The details of this technique are reported in our previous work.^26^

### Pure water permeability and rejection performance of individual membranes

**Fig. 9:**
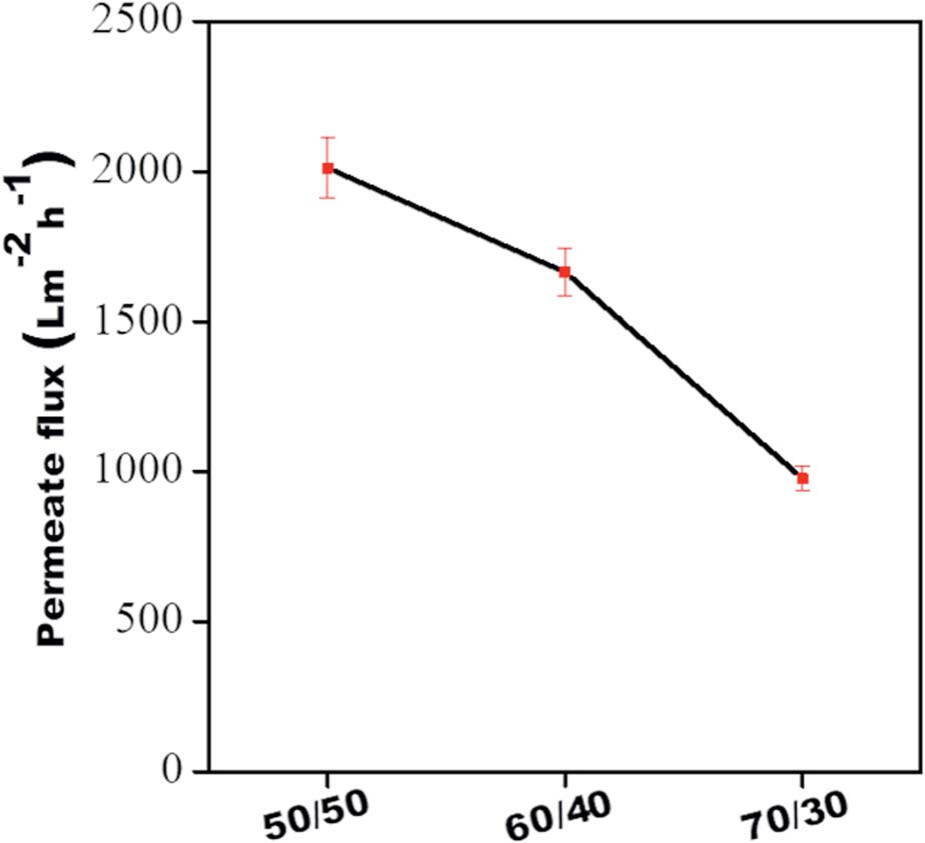
Pure water flux of all individual membranes.

Fig. 9 shows the pure water permeability of individual membranes at 60 psi. The pure water flux of all the individual membranes was 2010 ± 100 L m^−2^ h^−1^, 1665 ± 80 L m^−2^ h^−1^, and 976 ± 40 L m^−2^ h^−1^, respectively, for 50/50, 60/40 and 70/30. Though the membranes showed higher water permeability, their dye rejection performance was relatively low due to the larger pore size (as shown in Fig. 8), which allows the dye molecules to permeate through it quickly. Hence, to improve dye rejection performance, a unique approach was adopted here. The individual membranes were stitched with polyacrylic acid and referred to as control membrane. This also allows a gradient in morphology to be designed with largest pores as in 50/50 (PVDF/PMMA) at the top and the smallest pore as in 70/30 (PVDF/PMMA) at the bottom and pores with intermediate sizes sandwiched in between (as in 60/40, PVDF/PMMA blends). In addition, the surface of the control membrane was further modified *in situ* by a polyamide layer. To this end, a few membranes were designed wherein the free-standing GO membrane was physically stitched either with the control membrane without the PA layer in one case and with the PA layer in the other. The schematic representation of the gradient in morphology in the composite membranes is shown in Fig. 4. In order to fully understand the role of the free-standing GO layer towards improved dye and salt rejection, and excellent chlorine tolerance performance, GO was added *in situ* during the fabrication of the PA layer shown in Fig. 5.

### Spectroscopic evidence

**Fig. 10:**
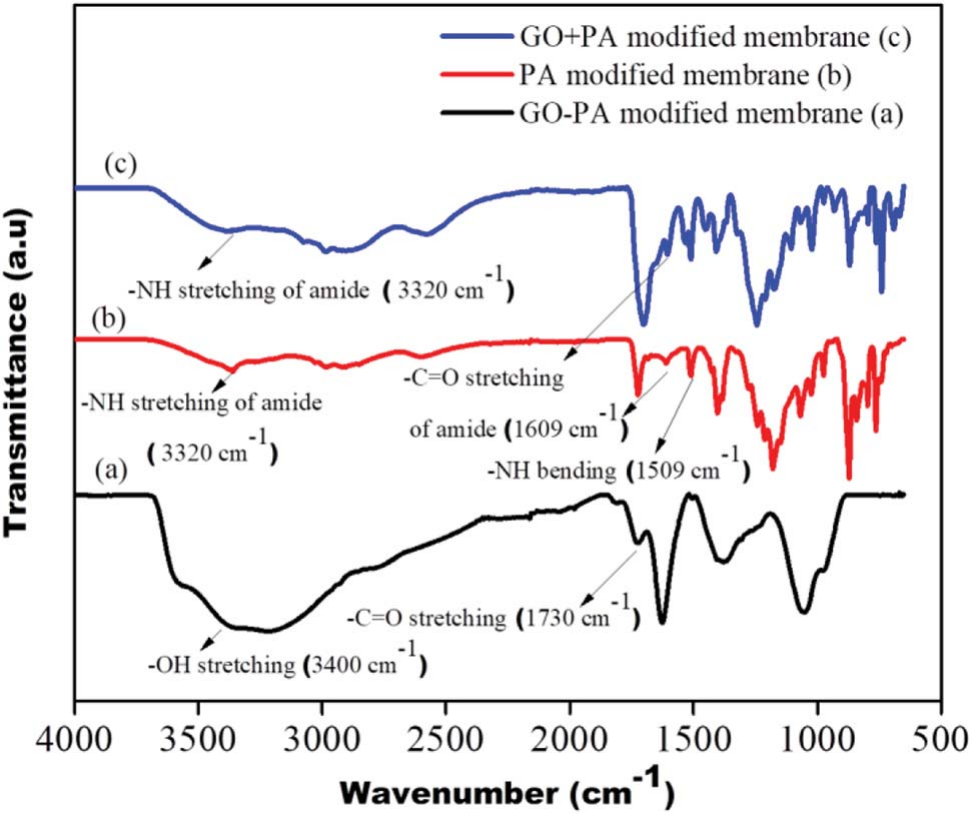
FTIR spectra of the GO–PA, PA and GO + PA modified membranes.

The GO + PA modification and PA modification on the control membrane's surface were confirmed using FTIR spectra shown in Fig. 10. The –OH stretching centred around 3300 cm^−1^ to 3500 cm^−1^, and –CO stretching at 1730 cm^−1^ suggests hydroxyl and acid groups in free-standing GO of the GO–PA modified membrane, respectively. The –NH stretching and bending at 3320 cm^−1^ and 1509 cm^−1^, respectively, and –C=O stretching at 1609 cm^−1^ confirm the amide layer's formation on the surface of the membrane. Thus, from the FTIR spectrum, it is understood that the polyamide layer and GO + PA layer are thriving on the membrane surface.

### Surface and cross-sectional morphology of the membranes

**Fig. 11:**
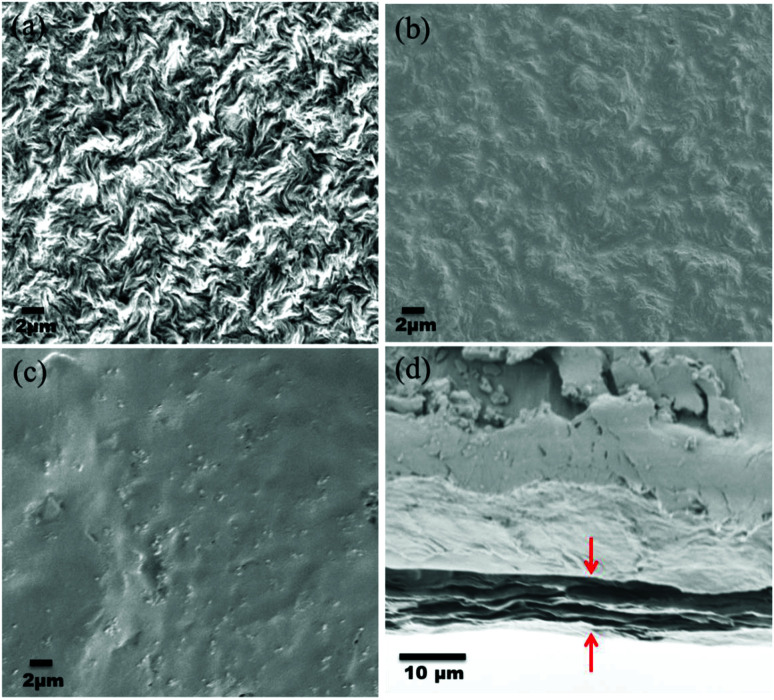
(a) SEM micrograph of the control membrane indicating spherulitic morphology, (b) surface morphology of the PA modified membrane, (c) surface morphology of the GO + PA modified membrane, and (d) cross-sectional morphology of the free-standing GO–PA modified membrane.

The deposition of the PA layer and GO + PA layer was confirmed using Ultra55 FESEM Carl Zeiss equipment. Fig. 11a shows the surface morphology of the unmodified membrane wherein spherulitic morphology is observed.^21^Fig. 11b and c show the PA and GO + PA modified membrane's surface morphology. From the cross-sectional morphology (Fig. 11d) of the GO–PA modified membrane, the thickness of free-standing GO is estimated to range from 7 to 8 μm and the overall thickness of the composite membrane lies in the range of 160–170 μm as shown in Fig. S4.†

### Contact angle measurement

The water contact angle is an important parameter to determine the hydrophilicity of the membrane qualitatively. In order to evaluate the hydrophilicity of each membrane, the static water contact angle was estimated using a contact angle goniometer. Generally, the lower the value of contact angle, higher will be the hydrophilicity and, therefore, the better affinity of water towards the surface of the membrane.^17^ The contact angle of the control membrane was 96 ± 3°. In contrast, the contact angles of the PA modified membrane, GO + PA modified membrane, GO modified membrane, and GO–PA modified membrane were 86 ± 2.5°, 63 ± 3°, 37 ± 1.2° and 38 ± 3°, respectively (Table 1). Thus, a drastic reduction in contact angle is contributed by the more hydrophilic surface in the modified membranes.

**Table 1:** Contact angle of various membranes used in this study

Sample	Figure	Contact angle (°)
Control membrane	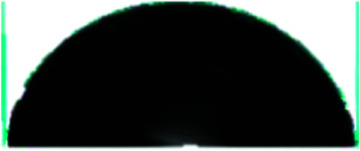	96 ± 3
PA modified membrane	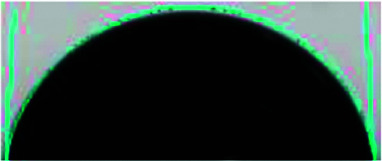	86 ± 2.5
GO + PA modified membrane	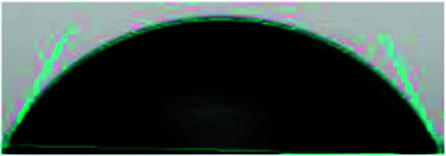	63 ± 3
GO modified membrane	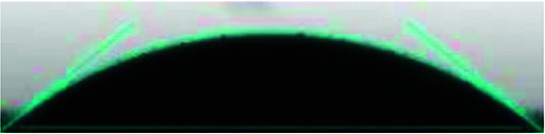	37 ± 1.2
GO–PA modified membrane	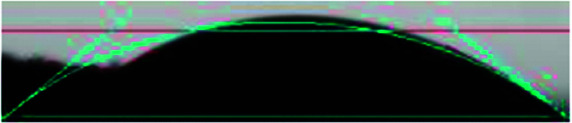	38 ± 3

### Pure water flux

**Fig. 12:**
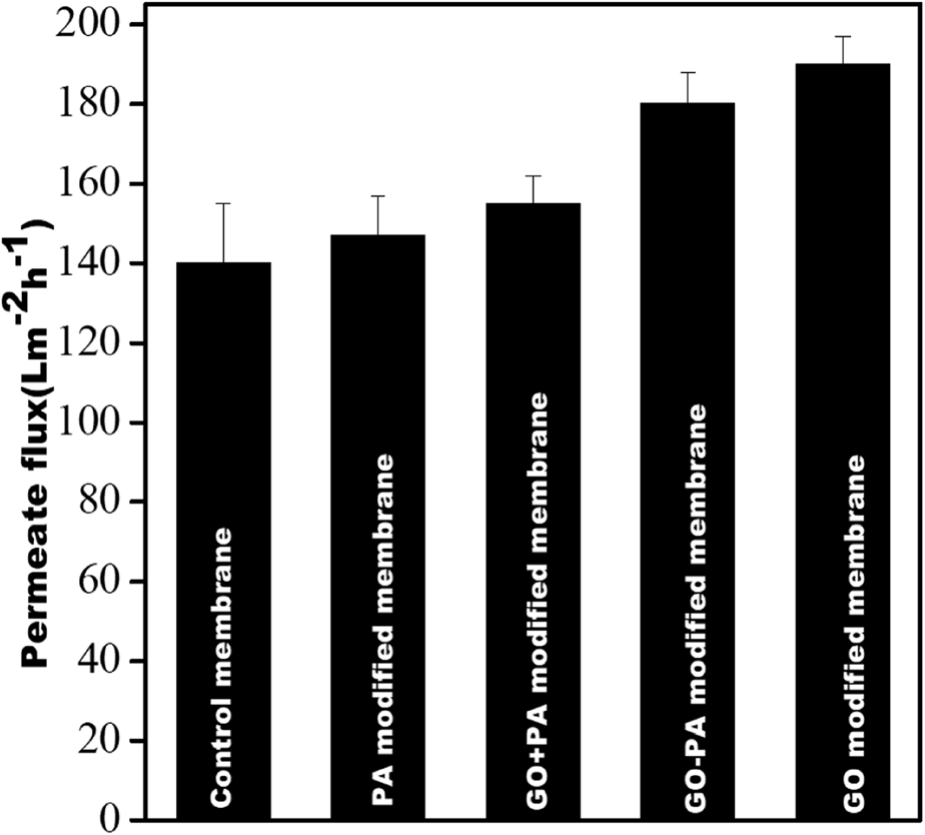
Pure water flux of all membranes @ 0.413 MPa.

Fig. 12 shows the pure water permeability of all the membranes at 0.413 MPa (60 psi). It was observed that the pure water fluxes obtained from the control membrane, PA modified membrane, GO + PA modified membrane, GO modified membrane, and GO–PA modified membrane were 140 ± 15 L m^−2^ h^−1^, 147 ± 10 L m^−2^ h^−1^, 155 ± 7 L m^−2^ h^−1^, 190 ± 7 L m^−2^ h^−1^, and 180 ± 8 L m^−2^ h^−1^, respectively. All four modified membranes showed higher water permeability due to the more hydrophilic nature of the membrane's surface. The GO modified membrane showed higher water permeability in comparison to the GO–PA modified membrane as free-standing GO enhances the water permeability through its channel due to being more hydrophilic. In contrast, permeability is reduced significantly due to reduction in pore sizes by deposition of the PA layer.

### Dye rejection studies

**Fig. 13:**
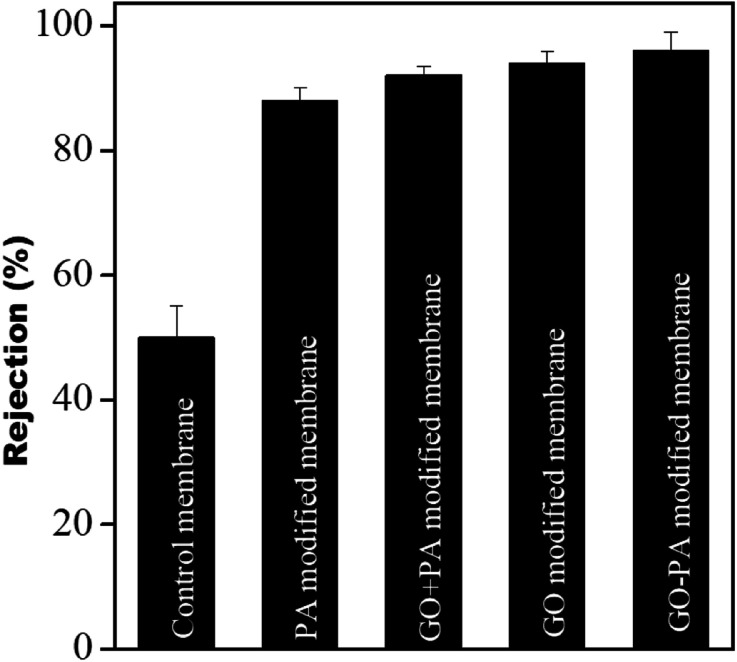
Dye rejection performance of the control membrane and different modified membranes.

The dye rejection of all the membranes was estimated using a 10 ppm feed of methylene blue as a cationic dye. It was observed that the rejection efficiency of all the modified membranes towards methylene blue was more than 90%, and was more than 96% for the GO–PA modified membrane. The control membrane showed a dye rejection of 50%; individual membranes failed to reject. The higher rejection efficiency in the modified membranes is mostly due to cationic dyes selectively adsorbing on the membrane surface due to electrostatic interaction and pore-based separation. The rejection efficiencies (%) obtained from the PA modified membrane, GO + PA modified membrane, GO modified membrane, and GO–PA modified membrane were 88 ± 2, 92 ± 1.5, 94 ± 3, and 96 ± 3, respectively. A comparison of various membranes is shown in Fig. 13.

### Stability of membranes

**Fig. 14:**
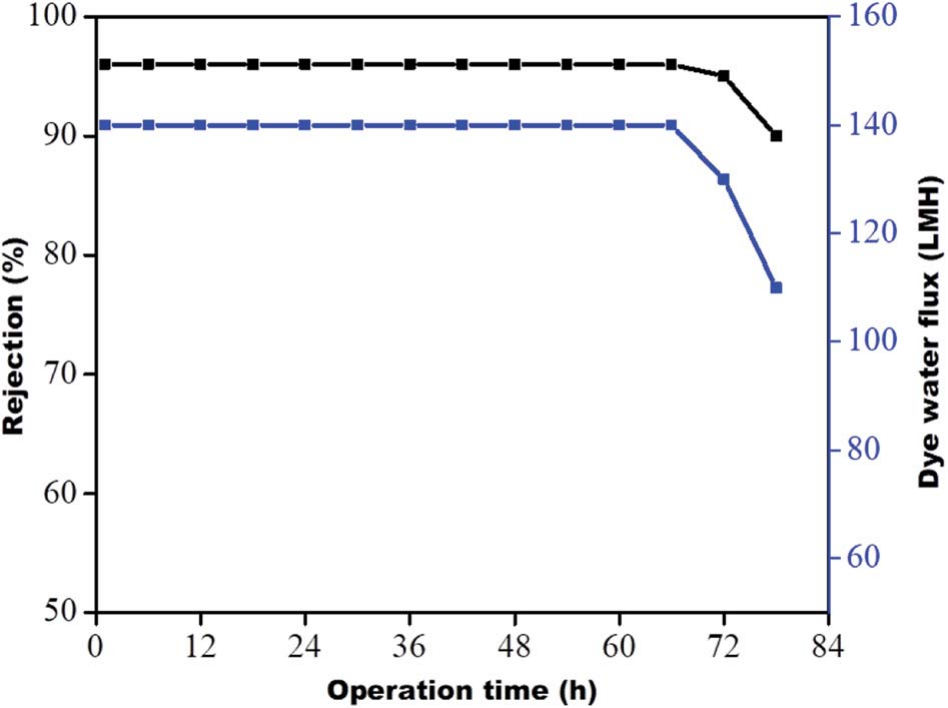
Long-term performance of the GO–PA modified membrane for 10 ppm methylene blue at 0.413 MPa.

The stability of the membrane was determined based on previous dye rejection and flux data by a procedure reported in the literature.^30,31^ The GO–PA modified membrane, which showed the highest rejection efficiency, was further examined *via* 78 h long-running at 0.413 MPa. The data were reported every six h. From Fig. 14, it is observed that the PA-GO modified membrane shows consistent rejection performance near about 96% for a period of 72 h without a significant decrease in rejection efficiency. The efficiency and flux are significantly reduced from 96 to 90% and 140 to 110 LMH, respectively, on further continuation of running. This is due to the adsorption of methylene blue (cationic dye) on the membrane's opposing surface, which may prevent further adsorption and hinder water permeance. Therefore, it may be concluded that the GO–PA modified membrane shows excellent stability without sacrificing its rejection performance.

### Dynamic antifouling studies

**Fig. 15:**
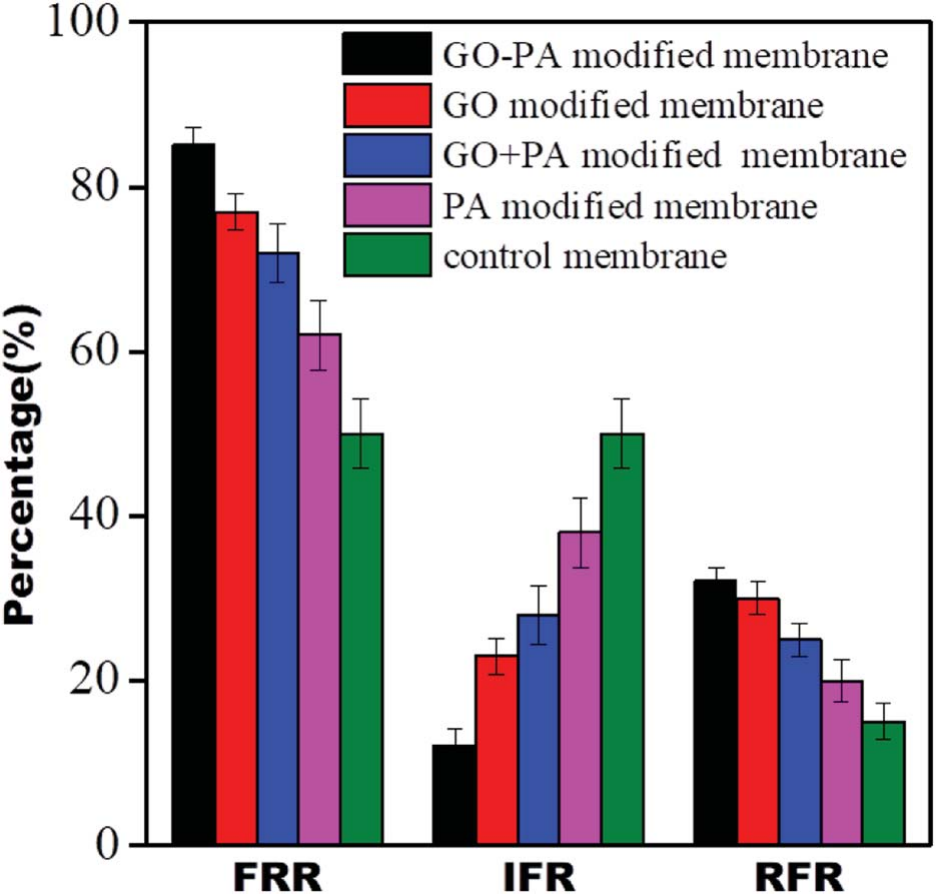
Dynamic antifouling studies for the control membrane and different modified membranes.

The antifouling properties of all the modified membranes were evaluated in terms of FRR, IFR, and RFR, shown in Fig. 15. It was observed that all GO modified membranes showed FRR more than 70%, and it was more than 85% for the GO–PA modified membranes. The GO–PA membrane allowed for higher rejection due to the negatively charged BSA's repulsion from the negatively charged free carboxylic and hydroxyl groups in the free-standing GO membrane.

### Desalination performance by FO

**Fig. 16:**
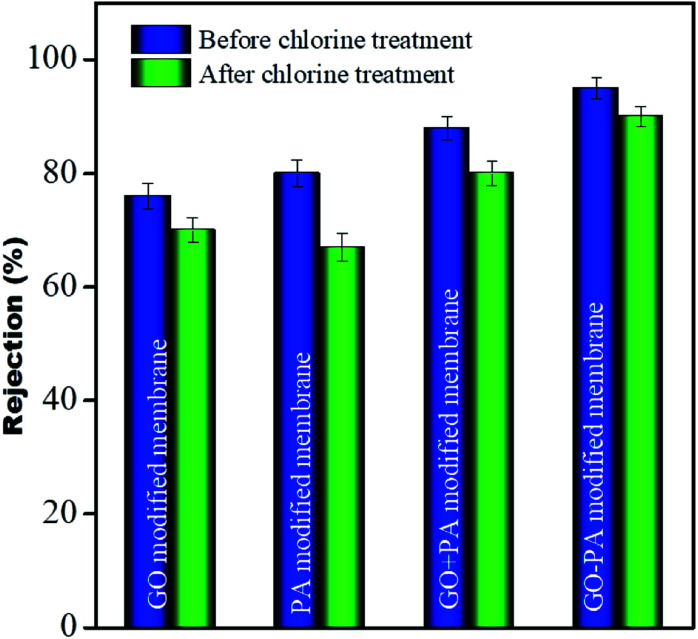
Salt rejection performance of all modified membranes.

The desalination performance of all modified membranes was assessed using pressure enhanced FO.^24^ DI water was used as feed and NaCl solution of 1000 ppm was taken as the draw solution for this experiment. The membrane was allowed first to compact for 30 min without applying any pressure to achieve a steady flow. The salt concentration on both feed and draw sides was monitored periodically at a pressure of 10 psi. No reverse salt flux on the feed side suggested no change in TDS on the feed side. The rejection for the GO modified membrane, PA modified membrane, GO + PA modified membrane, and GO–PA modified membrane was more than 76%, 80%, 88%, and 95%, respectively. The GO–PA modified membrane's highest efficiency of salt rejection is due to charge-based and pore-based sieving. In contrast, the control membrane exhibited only 22% rejection due to the larger pore size which fails to offer any resistance. The salt rejection performance of all the membranes is shown in Fig. 16.

### Chlorine tolerance performance

To investigate the chlorine tolerance property, all modified membranes were immersed in 2000 ppm NaOCl solution of pH 10 for 3 h. After washing all these membranes thoroughly with DI water these membranes were utilized for salt rejection performance. It was observed that the rejection for the GO modified membrane, only PA modified membrane, GO + PA modified membrane and GO–PA modified membrane after chlorine exposure was more than 70%, 67%, 80% and 90%, respectively. More than 13% decline in salt rejection was observed for the only PA modified membrane while only less than 6% and 8% decline in rejection was observed for the GO modified membrane and GO + PA modified membrane, respectively. And an excellent value of only less than 4% decline in rejection was observed for the GO–PA modified membrane. Thus, the GO–PA modified membrane showed excellent chlorine tolerability in comparison to the other modified membranes. The rejection performance is shown in Fig. 16.

### Antibacterial performance

**Fig. 17:**
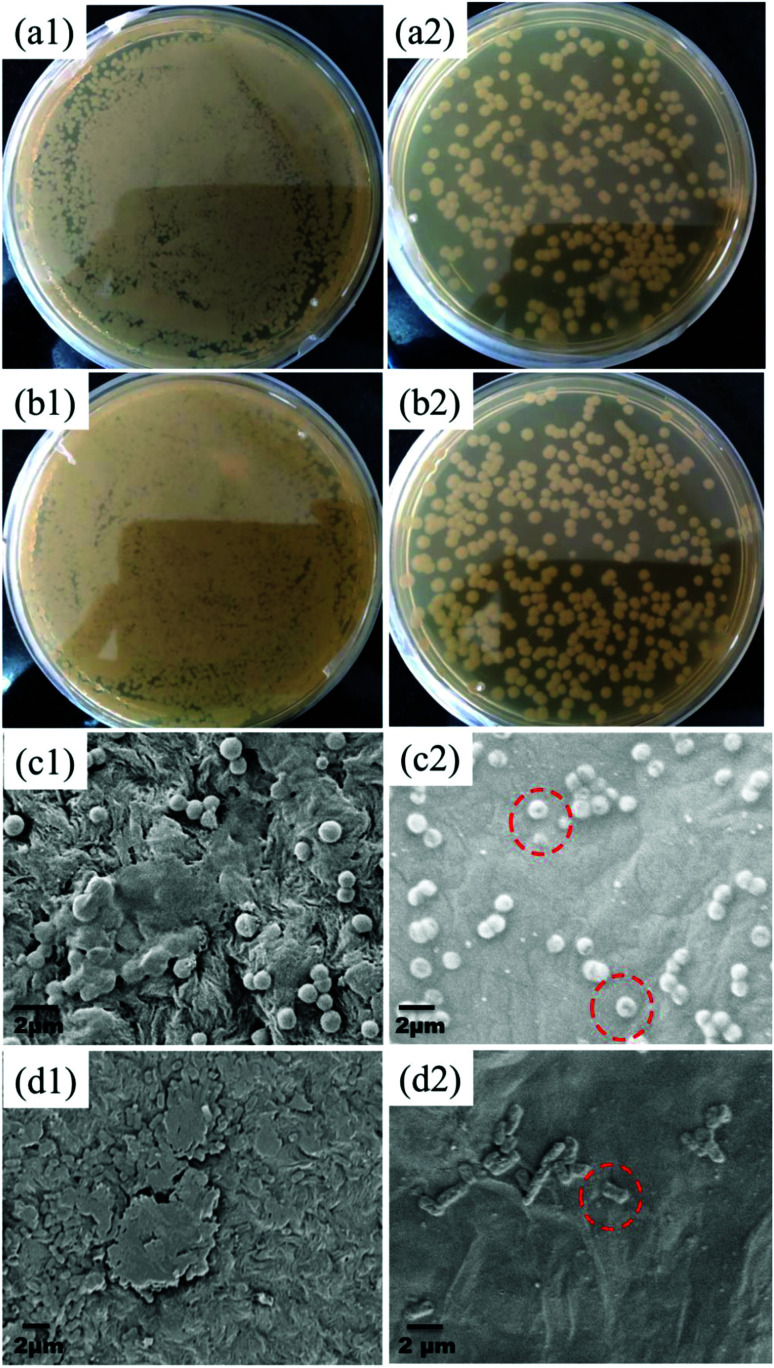
Digital images showing the CFU of *S. aureus* and *E. coli* incubated on the control membrane's (a1 and b1) and GO–PA modified membrane's surface (a2 and b2). Electron micrographs showing the surface of the control membrane (c1 and d1) and GO–PA modified membrane (c2 and d2) after two h of bacterial incubation with *S. aureus* and *E. coli*, respectively.

The GO–PA modified membrane's antibacterial efficiency was studied for both the bacterial strains through a standard plate count method. As seen from Fig. 17(a1)–(b2), it is noticed that there is a 2-fold reduction in the bacterial colonies for both strains for an incubation period of 2 h. Furthermore, to investigate the mechanism of antibacterial activity on the GO–PA modified membrane's surface, it was characterized by FE-SEM. The compromised structure of most of the bacterial cells was observed on the GO–PA modified membrane's surface (Fig. 17(c2) and (d2)), while the cells attached to the control membrane retained their original structure and formed a noticeable biofilm (Fig. 17(c1) and (d1)). These results proposed that the GO–PA modified membrane is antimicrobial, assisted by the effects against both the strains. The antibacterial mechanism of GO is based on the stress induced on the cell membrane by oxidative stress and direct physical interaction.^32–34^

## Discussion

In this work, three strategies were explored for size-selective sieving of ions, improved fouling resistance, and enhanced chlorine tolerance performance. In the first case, the free-standing GO layer was placed on top of the porous support without the PA layer. In the next case, the free-standing GO layer was placed on top of the PA layer formed *in situ.* Thirdly GO was mixed with the precursors to form GO + PA. A classical UCST system (PVDF/PMMA) was chosen here as the support layer. By selectively etching one of the blends' components, hierarchical porous structures with a gradient in pore sizes were obtained.

**Fig. 18:**
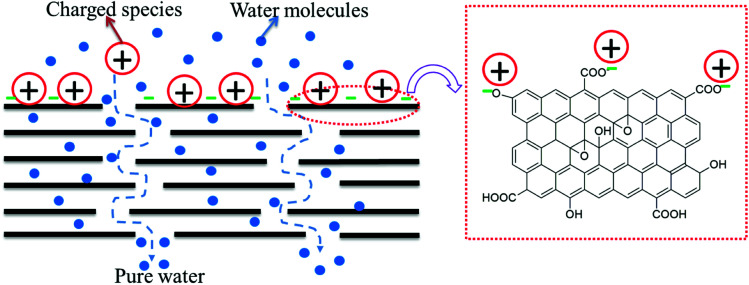
Schematic representation of the rejection mechanisms of GO.

In all three cases, excellent performance (in terms of permeability, rejection, antifouling, antibacterial activity, and chlorine tolerability) was observed, mainly contributed by the presence of GO on the surface of the membrane. The outstanding performance was observed when the templated support layer and a free-standing GO and PA layer in tandem were utilized. The nano-slit offered by GO would allow only water molecules to pass through and the charged species present on the surface of GO (COO^−^) would repel like charges and electrostatically bind with positively charged species. Hence, by geometrical restrictions and charge based sieving, GO can help in desalination. In membranes with a PA layer beneath the GO layer, any ions that pass through the flake structure of the GO layer would be arrested in the PA layer (see Fig. 18). Thus, the GO layer is responsible for the immediate rejection of salt and the secondary rejection is attributed to the fabrication of the PA layer beneath the GO layer.^35^ Moreover, PA is susceptible to chlorine attack and hence an additional GO layer would further protect the underlying PA layer, thereby improving the life of the membrane.

GO provides nanochannels within its lamellar structure through which water permeates very quickly, leading to more water permeability. Moreover, ions and dye molecules have to pass through this assembly of layers. The functional groups (like carboxyl, hydroxyl and epoxy) present in GO (as supported by the FTIR spectrum, shown in Fig. S2†), which are mainly hydrophilic, also play an essential role in improving permeability. These groups also contribute to the negative charge on the surface of the GO membrane (supported by the negative zeta potential reported in the literature^27^) which sieves the ions and dye molecules based on electrostatic interaction. The excellent fouling resistance of the GO–PA modified membrane is also due to the fact that the negative charge on the surface of the GO membrane repels the negatively charged BSA. The excellent chlorine tolerability of the GO–PA modified membrane is expected when GO is on the top. GO has phenolic moieties which can entrap the chlorine radical and form O–Cl thereby protecting the PA layer from chlorine attack.^21^ The GO layer on the top of the PA membrane also contributes to the antibacterial ability of the GO–PA modified membrane. The highly dispersive lamellar GO nanosheets show antibacterial mechanisms based on the stress induced on the cell membrane by oxidative stress and direct physical interaction as manifested from the antibacterial results.

## Conclusions

We exploited a unique concept in this work wherein a gradient in morphology was designed by stacking different porous membranes obtained by demixing a classical UCST blend, PVDF and PMMA, and selectively etching PMMA from the blend. This stack was stitched together by a suitable adhesive that doesn't clog the pores. Various strategies were explored in this study to achieve key attributes like desalination, antifouling, and chlorine tolerance. To selectively sieve ions and improve the chlorine tolerance performance, three different strategies were explored. In the first case, the free-standing GO membrane was used as the active layer. In the second case, the free-standing GO was positioned in tandem with the PA layer formed *in situ*. In the third case, GO was added during the formation of the active PA layer *in situ.* It was observed that the position of the GO layer controlled the desalination and chlorine tolerance performance in the membrane. For instance, when the free-standing GO membrane was positioned in tandem with the PA layer formed *in situ*, the salt rejection was more than 95% for a monovalent ion; fouling resistance was more than 85%; and dye rejection was more than 96% for a model cationic dye. This particular membrane showed excellent chlorine tolerance performance and antibacterial activity. This study will further help guide researchers working in this field from both academia and industry.

## Conflicts of interest

There are no conflicts to declare.

## Supplementary Material

NA-004-D1NA00513H-s001
