# Supplementary material for: Free-standing graphene oxide membrane works in tandem with confined interfacial polymerization of polyamides towards excellent desalination and chlorine tolerance performance
Source: Nanoscale Adv. 2021 Nov 19;4(2):467–78. doi: 10.1039/d1na00513h (PMC9418825; doi:10.1039/d1na00513h)
Supplement: NA-004-D1NA00513H-s001 [file NA-004-D1NA00513H-s001.pdf]

Electronic Supplementary Material (ESI) for Nanoscale Advances.
This journal is © The Royal Society of Chemistry 2021

Supporting Information For

Free standing graphene oxide membrane works in tandem with confined interfacial polymerization of polyamide towards excellent desalination and chlorine tolerance performance through Forward Osmosis.

Subhasish Maiti [a], Suryasarathi Bose*[a]

[a] Department of Materials Engineering, Indian Institute of Science, Bangalore, Karnataka, India-560012

*corresponding author: sbose@iisc.ac.in

Characterization of free-standing GO:

Structural properties of free-standing GO were analysed by Xpert Pro X-ray diffractometer using Cu Kα as a source. The free-standing GO was scanned from 5° to 80° with a step size of 0.049°. The XRD pattern of free-standing GO is shown in figure S1. The prominent peak at 10.4° is observed is corresponding to the oxidized graphitic peak.

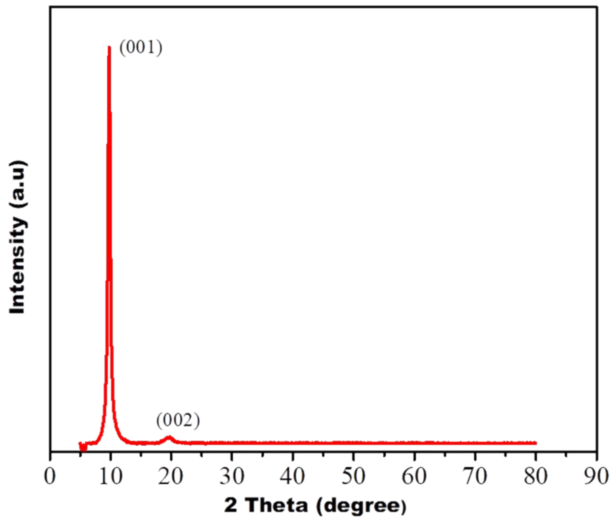

Figure S1: XRD pattern of free-standing GO.

The functional groups present in the free-standing GO were assessed using FTIR Spectra. From figure S2 it is clearly observed that peaks at 1720 cm^{-1}, 1625 $cm^{-1}$and 1050 cm^{-1} are characteristic peaks of carboxylic acid carbonyl stretching, aromatic C=C stretching and

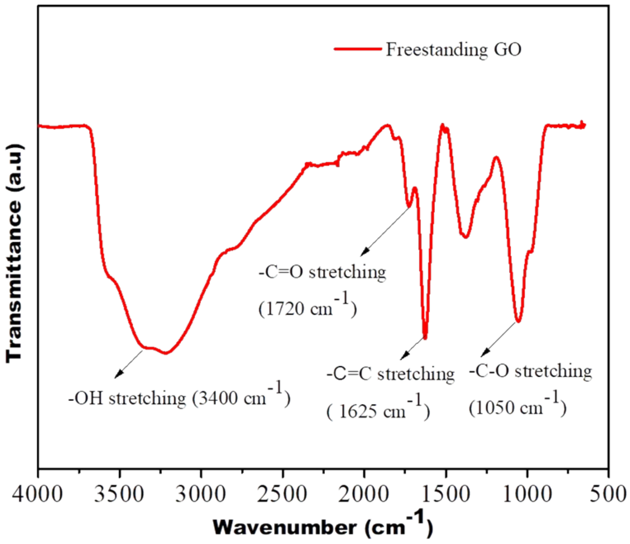

Figure S2: FTIR Spectrum of free standing GO.

epoxy C-O stretching respectively. Carboxylic acid group and hydroxyl groups are further validated by the appearance of an intense broad peak around 3400 cm^{-1}.

The surface and cross-sectional morphology of free-standing GO were characterized using a scanning electron microscope (SEM) by Ultra55 FESEM Karl Zeiss as shown in figure S3. From the cross-sectonal image it can be stated that thickness of free-standing GO is approximately 7-8 µm.

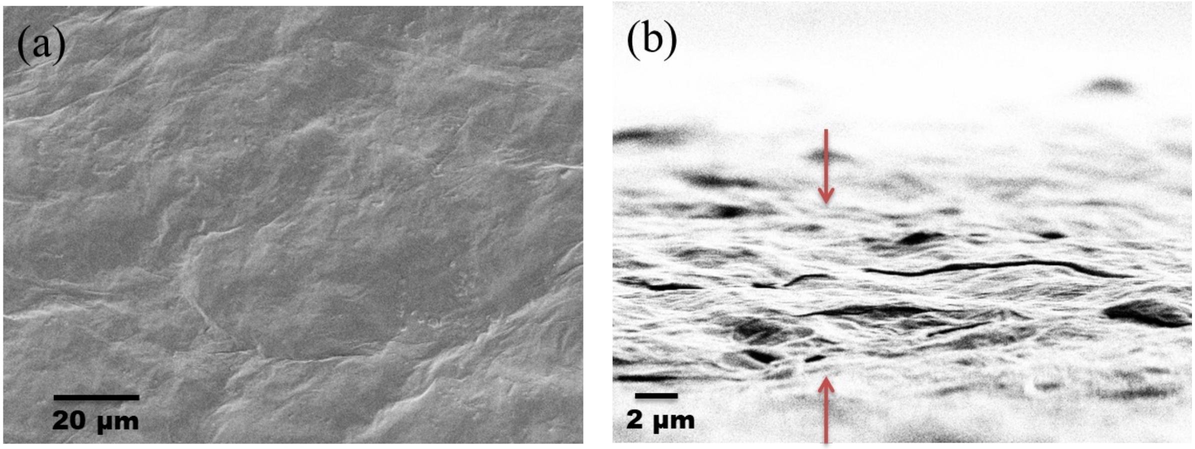

Figure S3: Micrograph showing the surface (a) and cross-sectional (b) morphology of free standing GO.

Cross-sectional morphology of composites membrane:

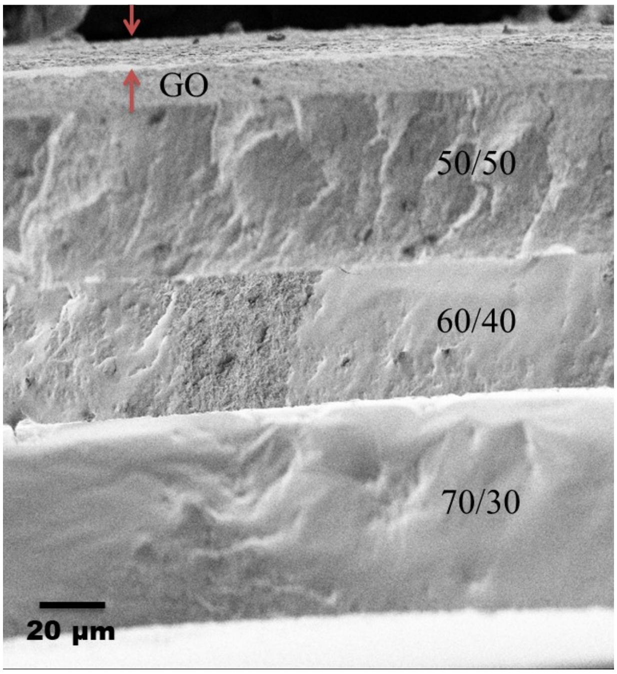

Figure S4: Micrograph showing the cross-sectional morphology of GO-PA modified composite membrane. It showing the overall thickness of the membrane in the range of 160-170 μm.
